# Gut Microbial Metabolite-Mediated Regulation of the Intestinal Barrier in the Pathogenesis of Inflammatory Bowel Disease

**DOI:** 10.3390/nu13124259

**Published:** 2021-11-26

**Authors:** Namrata Iyer, Sinéad C. Corr

**Affiliations:** 1 Department of Microbiology, Moyne Institute of Preventative Medicine, School of Genetics and Microbiology, Trinity College Dublin, Dublin, Ireland; IYERN@tcd.ie; 2 APC Microbiome Ireland, University College Cork, Cork, Ireland

**Keywords:** metabolome, microbiome, metabolites, inflammatory bowel disease, intestinal epithelium, gut barrier, metabolomics

## Abstract

Inflammatory bowel disease (IBD) is a chronic inflammatory disease. The disease has a multifactorial aetiology, involving genetic, microbial as well as environmental factors. The disease pathogenesis operates at the host–microbe interface in the gut. The intestinal epithelium plays a central role in IBD disease pathogenesis. Apart from being a physical barrier, the epithelium acts as a node that integrates environmental, dietary, and microbial cues to calibrate host immune response and maintain homeostasis in the gut. IBD patients display microbial dysbiosis in the gut, combined with an increased barrier permeability that contributes to disease pathogenesis. Metabolites produced by microbes in the gut are dynamic indicators of diet, host, and microbial interplay in the gut. Microbial metabolites are actively absorbed or diffused across the intestinal lining to affect the host response in the intestine as well as at systemic sites via the engagement of cognate receptors. In this review, we summarize insights from metabolomics studies, uncovering the dynamic changes in gut metabolite profiles in IBD and their importance as potential diagnostic and prognostic biomarkers of disease. We focus on gut microbial metabolites as key regulators of the intestinal barrier and their role in the pathogenesis of IBD.

## 1. Introduction

Inflammatory bowel disease (IBD) is a chronic inflammatory condition of the gastrointestinal (GI) tract that affects about 6.8 million people around the world, with an increasing rate of incidence in newly industrialized countries [1]. IBD has two main subtypes, Crohn’s disease (CD) and Ulcerative colitis (UC) [2]. While Crohn’s disease can involve any region of the digestive tract and tends to affect the entire intestinal wall, UC primarily affects the colon and is restricted to the inner lining of the intestine [3]. The aetiology of IBD is multifactorial with a combination of genetic and environmental triggers. Understanding the determinants of IBD onset, severity and relapse/remission are key to designing diagnostic and therapeutic strategies for disease management.

The contribution of genetics to IBD has long been recognized. Family members of IBD patients can have up to an 8–10 times higher risk of developing such diseases as compared to the general population [4]. However, this genetic component is stronger with CD as compared to UC. Studies with monozygotic twins show around 30–50% concordance for CD compared to only 10–15% in UC [4]. Genome-wide association studies (GWAS) have helped to provide insight into the genetic loci linked to IBD, with more than 200 loci identified thus far. Risk loci identified by GWAS are associated with key biological processes such as epithelial barrier function, epithelial repair, innate and adaptive immune response, autophagy, endoplasmic reticulum (ER) stress and metabolic pathways [2,4]. Loci such as the X-linked inhibitor to apoptosis (XIAP) and the interleukin (IL)-1 receptor gene were discovered in patients with early onset IBD [5]. While some prominent GWAS hits such as NOD2, ATG16L1 and IL23R have been well-studied, a functional insight into many of these loci is lacking since they occur in non-coding regions of the genome. This has resulted in a surge in research into non-coding RNAs, and other epigenetic mechanisms as regulators of IBD [6].

Genetics, however, explains only a fraction of IBD risk. The rising incidence of IBD in developing countries suggests a strong environmental component to the disease. Factors such as a high-fat diet, smoking, antibiotics and medication usage are all associated with increased risk of IBD [2]. The gut microbiota has emerged as a key player in inflammatory bowel disease. An imbalance in the gut microbial community composition is commonly found in IBD patients, with a trend towards the reduction of beneficial mucosa-associated bacteria such as *Faecalibacterium* spp. and a concomitant increase in Proteobacteria such as *Escherichia coli* [7]. The importance of the gut microbiota in disease pathogenesis has been observed; after antibiotic treatment or redirection of a faecal stream from an affected intestinal segment, an amelioration of inflammation in patients occurs [8]. While gut microbes are undoubtedly involved in the pathogenesis of IBD, it is unclear whether their role is causative or correlative. Dysbiosis could be the trigger for inflammation in susceptible individuals or could simply be a consequence of a dysregulated immune response in IBD patients. Understanding the mechanisms by which microbes trigger or contribute to the pathogenesis of IBD can help design microbiota-based therapies for disease management.

At the intersection of genetic, environmental, dietary, and microbial factors lies the intestinal epithelial layer. The epithelium forms the interface between gut microbes and dietary factors in the gut lumen and the immune cells present in the intestinal mucosa [9]. In this review, we will focus on the intestinal epithelium and explore its relevance in inflammatory bowel disease. In particular, we will elaborate on how metabolites produced by gut microbes can modulate the pathogenesis of inflammatory bowel disease via their effect on the intestinal epithelium.

## 2. Epithelial Barrier in Inflammatory Bowel Disease

### 2.1. Structure and Function of the Epithelial Barrier

The intestinal barrier is comprised of the intestinal epithelium and an overlying mucus layer. The epithelium is a single layer of intestinal epithelial cells (IECs) that forms a selectively permeable barrier that separates gut microbes and potential pathogens from mucosal immune cells and overall systemic circulation. This monolayer is made up of several different cell types and is organized into structures called crypts (large and small intestine) and villi (only in small intestine). Intestinal stem cells are located at the base of the crypts and undergo processes of proliferation and differentiation, giving rise to other cells in the epithelium, namely enterocytes, goblet cells, Paneth cells, microfold (M) cells, tuft cells and enteroendocrine cells [9]. Goblet cells secrete cross-linked mucin proteins that contribute to the formation of the mucus layer that overlays the epithelium while Paneth cells secrete anti-microbial peptides. Tuft cells are rare secretory cells that regulate type 2 immunity while M cells sample luminal antigens to prime microbe-specific immune response. The mucus layer contains anti-microbial peptides along with other factors such as immunoglobulin A, and restricts the growth and contact between luminal gut microbes and the epithelium [10].

One of the primary functions of the epithelium is nutrient absorption. This is achieved by the active absorption of nutrients by the epithelium (transcellular pathway) along with passive diffusion across the gaps between epithelial cells (paracellular pathway). Intercellular adhesion and communication in the epithelium are mediated by tight junctions, adherens junctions, desmosomes/hemidesmosomes and gap junctions. Of these listen junctions, tight junction proteins control the passive diffusion of solutes across the epithelium. The size and charge of tight junctions determines which molecules from the gut lumen are able to enter systemic circulation [11]. Paracellular permeability is tightly controlled via transcriptional and post-translational regulations of tight junction proteins. The cytokine tumor necrosis factor (TNF) α controls the activation of the myosin light chain kinase to upregulate the sodium ion passage through tight junctions. The upregulation of claudin-2 by cytokines such as TNFα, IL-13, IL-6, etc., also increases paracellular permeability [12]. Zonulin and zonulin-related proteins increase macromolecular or cellular movement across the epithelium by inducing reversible tight junction disassembly [13]. The activation of microbial sensors such as Toll-like receptor (TLR) 2 also regulates tight junctions to decrease barrier permeability [14]. Dysregulation of paracellular permeability can lead to the uncontrolled translocation of gut microbes and microbial antigens across the epithelium, leading to immune activation and inflammation [10].

Another challenge to the integrity of the gut barrier is epithelial turnover. The epithelium is completely renewed every 4–5 days [15]. The stem cells at the base of the crypt must continuously proliferate to achieve this renewal. Newly formed cells are pushed up from the crypt base along the crypt-villus axis, while old epithelial cells undergo shedding at the villus tip. Tight junction proteins such as occludin and zonula occludens-1 (ZO-1) are responsible for sealing off the gap left by the shedding epithelial cells [9]. Systemic exposure to microbial triggers, such as lipopolysaccharides (LPS) and concomitant increase in inflammatory cytokines such as TNFα, results in a marked increase in epithelial cell shedding and the loss of the barrier integrity [16,17]. Thus, the rate of epithelial proliferation, differentiation and shedding require carefully balanced to ensure that the integrity of the barrier is maintained.

### 2.2. Dysregulation of the Gut Barrier in IBD

The dysregulation of the epithelial barrier, aberrant microbial sensing and dysregulation of autophagy are common features in IBD patients. A microscopic examination of intestinal tissues from IBD patients reveals reduced goblet cell numbers, a defect in defensin production, disruption to the mucus barrier and an alteration in mucus composition [9]. Studies in animal models have revealed the importance of barrier integrity in the pathogenesis of IBD. A genetic knockout of the mucin protein Muc2 results in the spontaneous development of colitis in mice, attesting to the importance of barrier defects in disease onset [18]. The loss of Muc2 results in a compensatory upregulation of a goblet cell secretory factor Relm-β and anti-microbial Reg3γ. This further contributes to dysbiosis and increases the severity of colitis in these mice. Knockout of Relm-β in Muc2−/− mice attenuates the severity of colitis [19]. This suggests that the genetic mechanisms that affect the barrier and dysbiosis in the gut synergize to trigger inflammation in the gut.

Mouse models have provided functional insight into the regulation of the gut barrier via microbial sensing and autophagy pathways. Paneth cells are important secretory cells in the epithelium and are major producers of anti-microbials, such as defensins in the gut. Defensins protect the stem cells at the crypt base and assist in remodeling the gut microbiota composition [20]. Microbial sensing, autophagy and ER stress are all linked to the secretion of defensins. Mouse models of spontaneous ileitis (SAMP1/YitFc) show an increase in the number of abnormal Paneth cells. These abnormal cells experience ER stress, resulting in the secretion of misfolded defensins. This leads to dysbiosis and contributes to Crohn’s-like disease pathology [21]. These findings are supported by the observed reduction of defensin production in CD patients, suggesting that defects in Paneth cells can contribute to disease onset [22].

Increased intestinal permeability is observed in CD and UC patients, with higher permeability in symptomatic versus asymptomatic IBD and is related to disease symptoms such as diarrhea [23]. Serum and fecal levels of zonulin are also elevated in IBD patient cohorts compared to healthy controls [24,25]. The prevalent belief is that a leaky gut barrier results in aberrant exposure and translocation of gut microbes/microbial antigens to the intestinal immune system, triggering inflammation and, potentially, dysbiosis. However, most studies on intestinal permeability involve patients with diagnosed and ongoing IBD. It remains unclear if increased barrier permeability is a prerequisite for disease onset.

Animal models have provided insight into the role of barrier permeability in IBD onset. In the IL-10 knockout model of spontaneous colitis, an increase in barrier permeability precedes the onset of disease [26]. Similarly, mice lacking the xenobiotic transporter Mdr1 present with a dysregulation of tight junction proteins and barrier dysfunction, followed by spontaneous colitis [27]. However, knockout mice lacking the junctional adhesion molecule (JAM)-A or ZO-1 had an increased barrier permeability, but did not develop spontaneous colitis [28,29]. A mouse model overexpressing claudin-2 led to an increase in barrier permeability. Counterintuitively, this increased permeability and resulting microbial exposure triggered a tolerogenic immune response, increased epithelial proliferation and conferred protection against DSS-induced colitis [30]. These animal studies suggest that an increase in intestinal permeability might precede the onset of disease but may not be sufficient to cause disease.

Recently, a study by Turpin et al. investigated the causality of barrier permeability in IBD. They screened a cohort of asymptomatic, first-degree relatives of CD patients and found that an abnormally high intestinal permeability had a significant association with a future diagnosis of CD. Surprisingly, this association was observed even when the permeability test preceded the diagnosis by three years. This supports the hypothesis that increased barrier permeability is a precursor to Crohn’s disease and could be used as a biomarker to predict risk. It is noteworthy that 87% of the participants with abnormal permeability in their cohort remained asymptomatic during the study period [31]. This is in agreement with the findings observed in animal studies, suggesting that barrier dysfunction might be necessary but not sufficient for disease onset. Importantly, GWAS studies suggest that the contribution of genetics is relatively small, compared to environmental triggers for barrier permeability. The context in which an increase in barrier permeability occurs, and the microbial or environmental cues present, might determine whether the outcome is tolerance or susceptibility [9,32].

## 3. Gut Metabolites in Inflammatory Bowel Disease

### 3.1. Why Study Gut Metabolites in IBD?

Dysbiosis is well-documented in IBD patients and is primarily characterized by a loss of microbial diversity [33]. Most studies have documented the changes in microbiota during active disease, and relatively little evidence exists to support that dysbiosis in IBD occurs before disease onset [7]. Dysbiosis operates in a feedback loop with the intestinal immune response. An imbalance in the gut microbial community can exacerbate inflammation and, in turn, the inflammatory response creates conditions that can exacerbate dysbiosis. While definitive evidence has not yet emerged regarding whether dysbiosis can trigger the onset of IBD, it is nonetheless associated with disease severity [7]. This is supported by studies reporting the efficacy of faecal microbiota transplants (FMTs) as a treatment for IBD. In a Cochrane analysis of FMTs as a treatment for UC, it was found that FMT increases the rate of clinical remission in patients two-fold compared to controls, and remission was maintained for up to 12 weeks [34]. A pilot study assessed FMT as a strategy for the maintenance of clinical remission in CD. FMT recipients showed significantly higher rates of steroid-free remission at 10 weeks post-treatment, with lower disease severity scores at 6 weeks post-treatment [35]. These results suggest that the host–microbe interactions has significant implications for disease severity and progression. A shift in focus from microbial composition to microbial function could yield valuable insights into their role in IBD pathogenesis and better inform therapeutic strategies.

Microbes are very metabolically active in the gut. Microbes digest dietary fibres, synthesize vitamins and process host and environmental compounds. The metabolites made by microbes in the gut are actively or passively absorbed by the host and can enter systemic circulation [36]. Within the host, microbial compounds can be further modified by the host metabolism [8]. Comparisons between germ free mice and conventional mice highlight the extent of this metabolic exchange, for which up to 10% of plasma metabolites were significantly altered upon microbial colonization [37,38]. Microbial metabolite profiles in the gut are dynamic, reflecting the effects of changes in diet, microbial composition, microbial metabolism and host physiology. Metabolites in turn act as regulators of intestinal health via cognate receptor-mediated signalling in host cells or by activating host metabolic pathways.

Current diagnostics for IBD entail clinical, endoscopic, histological and biochemical tests. These rely on biomarkers that become apparent at a relatively advanced and symptomatic stage of the disease [8,39]. The changes in metabolite profiles in IBD have been documented using a variety of analytes such as stool, blood/plasma, urine and biopsy tissues [40]. Metabolomics holds the potential of being a more sensitive, non-invasive and potentially predictive biomarker that integrates information from environmental, microbial and host factors [41].

### 3.2. Metabolomics Techniques to Study Gut Metabolites

#### 3.2.1. Targeted and Untargeted Approaches

Technologies to visualize, identify, quantify, and analyze the complex array of metabolites produced in the gut have evolved significantly in recent years [42]. Metabolomics allows for the characterization of metabolite profiles across health and disease, a comparison of patterns and features to identify compounds of interest or cluster/classify disease states. Based on the hypothesis being tested, metabolomics analyses can either be targeted or untargeted. Targeted approaches are used when the metabolite or metabolite group of interest is already known. The target metabolite group dictates the choice of technique to maximize sensitivity, resolution, and quantification [43]. Untargeted approaches are the most common and help in the discovery of novel metabolites/features across groups. They can detect a wide variety of metabolite groups and are well-suited for high-throughput analyses. However, untargeted approaches suffer from lower resolution and complications in the identification of peaks [37]. We briefly summarize the design and execution of metabolomics analysis for IBD below. Full details on the technical aspects of metabolomics have been recently covered in other valuable reviews [42,44].

#### 3.2.2. Techniques

The most commonly used techniques for metabolomics are nuclear magnetic resonance (NMR) spectroscopy and mass spectrometry (MS). NMR is an unbiased, high-throughput and non-destructive analysis method. It involves easy sample preparation and does not require sample separation prior to detection. NMR can provide structural information about molecules but assigning identities to peaks can be challenging [8]. This is especially true if the sample is complex, since spectral overlap is hard to resolve in NMR. This can be partially overcome by two-dimensional acquisition [45]. Additionally, NMR has low sensitivity and is therefore more useful for identifying a higher concentration of metabolites in the sample.

Mass spectrometry lends itself well to the high complexity of biological samples. Samples can be analyzed via direct injection or can be coupled to an online separation platform such as liquid or gas chromatography. Separation systems provide additional resolution power and additional chemical information based on retention time. MS can be very versatile, presenting choices in upstream (GC/LC/UHPLC) and downstream (MS-MS) platforms, ionizers, and detectors. MS also has the advantage of higher sensitivity, specificity, and a more dynamic range in relation to metabolite concentrations [46]. GC-MS is the method of choice for volatiles (e.g., short chain fatty acids; SCFAs) while LC-MS is suited for polar/non-polar metabolites. However, MS requires additional derivatization of the sample before analysis which can be cumbersome for high-throughput analyses [45].

#### 3.2.3. Sample Types and Sample Processing

There is no single metabolomic method that is able to provide the full spectrum of metabolites in any sample alone. Sample processing methods are selected with respect to each specific class of metabolites. For example, the choice of either methanol/methanol-water versus chloroform/hexane as a solvent depends on polar or lipophilic metabolites, respectively [45]. A combination of different processing methods and metabolomics platforms can provide a more comprehensive picture of the metabolites in the sample [37,41].

Additionally, the choice of analyte can determine the type of biological insight the analysis will provide. Faecal samples or faecal water samples are a very common analyte and are rich in information about intestinal function and microbial activity in the gut. However, microbiota composition and their cognate metabolites vary significantly along the GI tract [47]. Faecal samples, therefore, are not necessarily representative of metabolites in other biogeographical locations within the gut [48,49]. Faecal samples are also downstream of intestinal absorption. Some metabolites are absorbed or utilized as an energy source by intestinal epithelial cells [50], affecting their final concentration in the faecal samples. Intestinal absorption, digestion and other host processes are also affected in IBD [51], which in turn might affect the faecal sample [8]. Additionally, drug treatments could be confounding factors by virtue of introducing exogenous metabolites that might interfere/overlap in spectral regions of crucial metabolites [52]. Other analytes such as serum or plasma give information about gut metabolites (of dietary, microbial and host origin) that have been absorbed into the system. Some of the microbial metabolites are subject to modification by host metabolic processes after absorption which can complicate the metabolomics analysis [53].

#### 3.2.4. Data Pre-Processing and Analysis

Once spectra for all the samples are acquired, data is subjected to pre-processing steps such as filtering, calibration, spectral alignment, and normalization before different samples can be compared. Data is analyzed using supervised (e.g., principal component analysis, PCA) and unsupervised (e.g., partial least squares discriminant analysis; PLS-DA) statistical tools to find discriminatory features or biomarkers in a sample set [45]. Further, databases such as human metabolome database (HMDB), METLIN or ChemSpider can be used to identify metabolites from their spectral signatures [54]. However, despite database searches, many metabolites still remain unidentified. This introduces a bias in metabolomics studies towards well-annotated and high concentration metabolites in the sample [42,45].

### 3.3. Gut Metabolome Changes in IBD

#### 3.3.1. Overview of Clinical Studies

Metabolomics investigations in IBD span several different analytes from plasma/serum, urine, faeces, intestinal biopsy to breath analysis [40]. Across different studies, metabolomics has successfully differentiated between healthy and IBD samples. However, efforts to differentiate between IBD sub-types, CD and UC, have had mixed success [37,55]. This is partly due to the inherent heterogeneity among CD and UC patients in terms of disease severity, treatment and surgical history and current status of disease (active flare/remission). In addition, relatively few studies have looked at metabolomics to monitor the treatment response of IBD patients [45,56].

Serum and plasma metabolomics report changes in branched chain amino acids (BCAA) such as leucine, isoleucine and valine in CD and UC patients. Isoleucine was elevated while leucine and valine were reduced in CD and UC patients. BCAA reduction is associated with a concomitant increase in its breakdown product 3-hydroxybutyrate [40,57]. Glutamine is consistently reported as lower in IBD patients, and notably lower in CD patients compared to UC [57,58]. Histidine is also reported to be lower in IBD patients and might potentially be a biomarker for relapse in UC patients [59]. A study with a large German cohort of IBD patients also reported a strong negative correlation between serum tryptophan (Trp) levels and disease activity. Lower serum tryptophan in IBD was accompanied by an increase in its metabolite quinolinic acid, suggesting Trp degradative activity in IBD [60].

Metabolomics on urine samples reveal a reduction in hippurate in IBD patients. Hippurate is a host-microbial co-metabolite, where dietary phenols are converted to benzoate by microbes and then to hippurate in the host liver [53]. Additionally, compounds such as formate, trigonelline (niacin metabolite), TCA intermediates (citrate and succinate) and SCFAs (acetate, 2-hydroxyisobutyrate and butyrate) are all reduced in urine samples of IBD patients compared to healthy controls [40,61].

Stool metabolites have been extensively studied in IBD patients. Overall, the metabolite diversity in IBD patients is lower than controls and mirrors the loss of microbial diversity in the IBD gut [62,63]. Metabolite diversity is also affected by physiological factors such as poor nutrient absorption, increased fluid content in the bowel and shortened transit times through the gut [64]. IBD patients tend to have lower faecal levels of short and medium chain fatty acids, secondary bile acids, sphingolipids and vitamins [65]. This is concomitant with an increase in primary bile acids, amino acids, polyamines, arachidonate and acylcarnitines in IBD [66]. Of these, the interpretation of amino acid and bile acid data is complicated by potential disease-induced malabsorption as a confounding factor [37,52]. Lloyd Price et al. make a useful observation in their longitudinal study regarding periods of dysbiosis in their patient cohort (mix of active and remission patients). In their study, dysbiosis was characterized by a loss of obligate anaerobes, an increase in facultative anaerobes and correlated with high variability in their multi-omic dataset [64]. Their observation highlights the complexity of metabolomic analysis in IBD and underscores the need for multi-omic approaches, comprehensive metadata collection and larger cohort sizes. Results of recent untargeted metabolomics studies of faecal samples in IBD are summarized in Table 1.

#### 3.3.2. Faecal Metabolomics as a Diagnostic or Prognostic Tool for IBD

Differentiation between sub-types and disease states in IBD has been a primary objective of several metabolomics studies with mixed results [57,67,68]. Marchesi et al. used NMR-based metabolomics to reveal significant differentiation between IBD and healthy controls, as well as between CD and UC patients. They observed a reduction in butyrate, acetate and increased amino acids in CD faecal samples as effective discriminators against UC [3]. On the other hand, in an analysis of faecal metabolomes, conducted by Bjerrum et al., the effective discrimination between UC and CD samples was complicated by patient medical history such as intestinal surgery and anti-TNF therapy [69]. To avoid some of the confounding factors introduced by treatments, Kolho et al. performed a metabolomics analysis in a newly diagnosed, treatment-naïve cohort of pediatric IBD patients. They found that UC faecal samples consistently had higher levels of metabolites such as amino acids, kynurenine, taurine, creatinine, and normetanephrine compared to CD and healthy controls. Using faecal metabolomics, they observed an effective discrimination between UC and CD patients [70].

Using GC-MS, De Preter et al. identified medium chain fatty acids (MCFAs) as discriminators of IBD. Furthermore, they identified hexanoate and styrene as correlates of disease severity in CD and UC patients, respectively [71]. Similar trends in SCFAs and MCFAs were also observed in other studies [72,73]. The study by Ahmed et al. effectively discriminated between active CD and active UC, however, failed to discriminate between active and inactive UC patients [72]. By analyzing dietary preferences, microbiome and metabolomics data, Weng et al. identified correlations between these variables in the IBD gut [73]. A multi-omic analysis in healthy, asymptomatic relatives of pediatric IBD patients helped to identify IBD-like microbial and metabolic features, suggesting that microbiome and metabolome imbalances might contribute to the risk of IBD onset [74].

**Table 1:** List of studies performing untargeted comparative faecal metabolomics in IBD.

Study	Patient Groups	Metabolomics Technique	Upregulated Metabolites	Downregulated Metabolites
Le Gall et al., 2011 [52]	UC (*n* = 13), IBS (*n* = 10), Healthy (*n* = 22)	^1^H NMR	**In UC:** Taurine, Cadaverine, Glucose and Choline	**In UC:** 2-methylbutyrate
Walton et al., 2013 [55]	Healthy (*n* = 19), CD (*n* = 22) and UC (*n* = 20)	GC-MS		**In CD versus healthy:** Butanoic aid, 1-propanol, propanoic acid, butanoic acid, indole
Bjerrum et al., 2015 [69]	UC (*n* = 48), CD (*n* = 44), Healthy (*n* = 21)	^1^H NMR	**In active CD and active UC:** Amino acids **In active UC:** Lactate, Taurine	**In active CD:** Butyrate and Propionate
De Preter et al., 2015 [71]	Healthy (*n* = 40), CD (*n* = 83), UC (*n* = 68)	GC-MS	**In CD:** 1-ethyl3-methylbenzene, benzene acetaldehyde, phenol, 2-methyl propanal, carbon disulfide and 1-methoxy-4-methylbenzene **In UC:** cyclohexane, 3-methyl butanal and pyrrole	**In CD and UC:** Medium chain fatty acids, Protein fermentation metabolites **In UC only:** Furan, 5-methyl-2-furancarboxaldehyde and 3,4-dimethylthiophene
Jacobs et al., 2016 [74]	Paediatric IBD patients (*n* = 36), Healthy family (*n* = 56)	UPLC- ToFMS	Amino acid derivatives and bile acids	Stercobilin, Acetyl-glutamic acid and boldione
Ahmed et al., 2016 [72]	Healthy (*n* = 109), CD (*n* = 117) and UC (*n* = 100)	GC-MS	**In active CD:** 1-octen-3-ol, heptanal, propanal, benzeneacetaldehyde, 6-methyl-2-heptanone and decane	**In active CD:** Pentanoic acid, 2-methyl butanoic acid, methanethiol, 3-methylphenol
Santoru et al., 2017 [62]	Healthy (*n* = 51), UC (*n* = 82) and CD (*n* = 50)	1H NMR, GC-MS and LC-QToF-MS	**In CD:** Alanine, phenylacetic acid, glyceric acid, phenylethylamine, putrescine and cadaverine, diacylglycerols **In UC:** Cadaverine, alanine, 4-hydroxyphenylacetic acid, 4-aminovaleric acid, TMAO, diacylglycerols	**In CD and UC:** Vitamins, 3-methyladipic acid, 5β-coprostanol, 3-hydroxybutyric acid, 2-hydroxy-3-methyvaleric acid and hydrocinnamic acid, urobilinogen
Kolho et al., 2017 [70]	Healthy (*n* = 14), IBD (*n* = 23)	UPLC-MS/MS	**In UC:** Amino acids, citrulline, ornithine, creatinine, choline, kynurenine, taurine, normetanephrin	**In UC:** Cytosine
Alghamdi et al., 2018 [56]	Healthy (*n* = 11) and CD (*n* = 11)	LC-MS	C20 Sphingenine, octadecenoylsphingenine, Sphingomyelins, LCFAs	Ornithine isomer, Tyrosine
Weng et al., 2019 [73]	Healthy (*n* = 42), UC (*n*= 107) and CD (*n* = 173)	GC/MS, LC-NEG/MS, and LC-POS/MS		**In UC and CD:** LCFAs, MCFAs, bile acids, and vitamins **In UC versus healthy:** Glycochenodeoxycholate and glycolithocholic acid
Franzosa et al., 2019 [63]	Healthy (*n* = 34), CD (*n* = 68), UC (*n* = 53)	LC-MS (combination of 4 techniques)	**In CD versus healthy:** Sphingolipids, carboximidic acids, bile acids, lactate	**In UC and CD:** Triterpenoids and LCFAs, triacylglycerols, pantothenate
Lloyd Price et al., 2019 [64]	132 subjects (non-IBD, UC and CD)	LC-MS (combination of 4 techniques)	**In CD versus non-IBD:** Polyunsaturated fatty acids, Nicotinuric acid, Bile acids, acylcarnitine	Vitamins, Lithocholate and deoxycholate, SCFAs

Abbreviations: Ulcerative colitis (UC); Crohn’s disease (CD); Inflammatory bowel disease (IBD); Proton Nuclear magnetic resonance spectroscopy (^1^H NMR); Liquid chromatography Mass spectrometry (LC-MS); Gas chromatography Mass spectrometry (GC-MS); Quadruple time of flight (QToF); Ultra-Performance Liquid Chromatography (UPLC).

Santoru et al. observed that CD and UC samples increased the abundance of amino acids, polyamines, and diacylglycerols. The authors observed a decrease in vitamins, 3-hydroxybutyric acid, 2-hydroxy-3-methyvaleric acid, and urobilinogen in CD and UC samples compared to healthy controls. Additionally, UC samples also showed an increase in trimethylamine-N-oxide (TMAO), tyramine and 4-aminovaleric acid. A PLS-DA analysis showed a clear distinction between healthy controls and CD/UC. However, they found significant overlap between CD and UC metabolite profiles, making a distinction difficult [62]. Interestingly, another study observed an increase in TMAO in plasma samples of UC patients compared to controls [75]. A significant overlap between UC and CD samples was also observed in the study by Franzosa et al. Interestingly, they found UC samples showed diffused distribution, with some having more control-like and others more CD-like metabolomes. This distribution showed a positive correlation with their level of inflammation as measured by faecal calprotectin and interfered with effective discrimination of UC and CD samples [63]. Overall, studies with a large cohort size, showing effective discrimination between UC and CD using metabolomics are relatively few. This suggests that inherent heterogeneity in UC and CD patients and smaller cohort sizes limit the effectiveness of metabolomics as a diagnostic tool.

Metabolomics has recently also been employed as a tool to assess the response of patients to therapy. Approximately 25% of CD patients undergo anti-TNF therapy, among which almost a third of the patients will not have sustained response to treatment [76]. Using NMR analysis of faecal metabolites in CD patients, Taylor et al. showed that patients who responded to therapy had significantly higher levels of valerate while non-responders had higher levels of lysine [77]. Metabolites associated with anti-TNF-induced remission were also evaluated via longitudinal analysis in a German cohort of adult IBD patients. At the microbiome level, no significant difference was present in the communities of remitters and non-remitters, however faecal metabolome analysis revealed that butyric acid was significantly increased in remission patients while non-remitters showed an increase in 3-methyl-thiopropionic acid and methyl 2-(methylthio) acetate [78]. These studies provide insight into the crucial microbe-metabolite relationships that underlie therapeutic success and provide avenues for the better design of therapeutic strategies.

Markers that provide prognostic information about treatment response are particularly valuable in deciding the best course of treatment for a patient. In a cohort of newly diagnosed pediatric CD patients, Wang et al. uncovered that patients with higher faecal levels of L-aspartic acid, linoleic acid and L-lactic acid at baseline showed a sustained response to treatment. Conversely, patients who did not show a sustained response had higher baseline levels of N-acetylserotonin, methylglutaric acid, adipic acid, 4-aminohippuric acid, and isovaleric acid in their faecal samples [79]. Studies have identified bile acid metabolites in both serum and faeces as significant prognostic biomarkers of treatment response [80,81]. The dominance of primary bile acids in faecal samples (>70% of total bile acid pool) was also found to be a negative predictor for a sustained response to nutritional therapy in a study of pediatric CD patients [82]. This suggests that metabolite biomarkers have the potential to outperform clinical markers such as faecal calprotectin or C-reactive protein as predictors of anti-TNF therapy response [83].

## 4. Gut Metabolites as Key Regulators of Epithelial Barrier Function in IBD

Gut metabolites change rapidly in response to environmental factors, even in the absence of a change in the microbiota composition [84]. Microbial metabolites act as signalling molecules in host–microbe communication. The intestinal epithelium is the primary interface of host–microbe interactions and are directly affected by microbial metabolites. IECs regulate the absorption of these metabolites, use them for energy metabolism and possess cognate receptors that modulate epithelial functions in response to metabolites. The importance of metabolites in regulating the epithelium and overall intestinal immunity has been emphasized by recent studies highlighting the ability of sterile faecal filtrates being sufficient to mediate the beneficial effects of whole microbial communities [85]. Of the complex array of metabolites produced by microbes, very few have been functionally characterized. In vitro and in vivo models (including gnotobiotic mice, genetic and chemical models of IBD) have been very useful for providing functional insight into the role of the microbiota and their metabolites in IBD [86,87,88]. However, animal models do have their limitations. A meta-analysis revealed that animal models of colitis only share about 17% of the differentially regulated metabolites with human studies of IBD [89]. Insights from animal models on the prominent classes of gut metabolites, their microbial producers, host receptors and effects on the intestinal epithelium are summarized in Table 2.

### 4.1. Short Chain Fatty Acids

Short chain fatty acids (SCFAs) are the metabolic by-products of microbial fermentation of complex polysaccharides or fibres. Of the different fibre types, soluble fibres produce a higher SCFA yield compared to non-soluble fibres [90]. Acetate, propionate and butyrate comprise almost 95% of the total SCFAs in the gut and are present at a ratio of 60:20:20 in the mouse and human gut [46]. Other SCFAs produced in the gut include branched chain fatty acids (isobutyrate, 2-methylbutyrate and isovalerate), lactate, succinate, formate, caproate and valerate. SCFAs can reach concentrations of up to 13 ± 6 mmol/kg content in the distal ileum and 80 ± 11 mmol/kg in the colon [90].

SCFAs are primarily synthesized by obligate anaerobes in the gut. Butyrate producers belong to clostridial clusters I, III, IV, XI, XIVa, XV and XVI, which include *Faecalibacterium prausnitzii* and *Roseburia intestinalis* [90]. Acetate is synthesized by members of *Bacteroides* spp., and *Prevotella* spp. *Bacteroides* spp., *Veillonella* spp., *Dialister* spp. and *Ruminococcus* spp. are major producers of propionate [91]. The reduction in SCFAs in IBD faecal samples is correlated to a loss of microbial species such as *F. prausnitzii* and *R. homonis* [40].

SCFAs, especially butyrate, act as an important source of energy for colonocytes and are actively taken up by IECs via transporters such as monocarboxylate transporter-1 (MCT-1). SCFAs also bind to G-protein coupled receptors (GPCRs) such as GPR43, GPR41 and GPR109A to activate cellular signalling pathways and modulate epithelial function [92]. SCFA-sensing by GPR43, for example, drives the expression of anti-microbial peptides Reg3γ and β-defensins in the intestinal epithelium [93]. The beneficial effects of dietary fibre in the dextran sulphate sodium (DSS) model of colitis, are mediated in part by GPR43. Microbial lactate activates GPR81 (expressed on Paneth cells) and activates downstream Wnt signalling to promote epithelial regeneration [94]. The microbial metabolite succinate drives the expansion of Tuft cells in the small intestine, which suppress type 17 response to confer protection against inflammation [95]. Additionally, butyrate and propionate can inhibit class I histone deacetylase (HDAC) activity, thereby increasing histone acetylation and promoting the regulation of cellular transcriptional activity. This HDAC inhibitory activity is responsible for suppression of epithelial proliferation by propionate and butyrate [91].

SCFAs promote the integrity of the epithelial barrier. Butyrate upregulates the expression of tight junction proteins such as occludin and claudin-1 on epithelial cells via the AMP-activated protein kinase (AMP-K) pathway [96]. Furthermore, via HDAC inhibition, butyrate promotes synaptopodin expression, which is involved in tight junction formation and maintenance [97]. Butyrate also regulates mucin expression in epithelial cell lines [98,99,100]. Butyrate can also counteract the increase in barrier permeability induced by inflammatory cytokines [101]. However, there is some evidence to the contrary in primary cell models from IBD patients [102]. Another study suggests that butyrate might even synergize with TNFα to promote inflammation [91]. Due to its barrier protective properties, increasing luminal butyrate has been suggested as a potential treatment for IBD patients. However, a study on epithelial cell metabolic capacity in IBD patients suggested that inflammation reduces the ability of the epithelium to consume butyrate [103]. Thus, in IBD a loss of butyrate producing species occurs, as well as a lowering of host responsiveness to butyrate due to inflammation.

### 4.2. Tryptophan Metabolites

Tryptophan (Trp) is the substrate involved in several host and microbial transformations. It is metabolized via the following three major pathways: Kynurenine (Kyn) pathway in the host, the indole pathway by gut microbes and the hydroxytryptamine (HT) pathway in a type of enteroendocrine cell called enterochromaffin cells. Each pathway has distinct metabolic products that can affect intestinal function [46]. Microbes such as *Lactobacillus* spp., *Clostridium* spp. and *Bacteroides* spp. express the enzymes necessary for tryptophan biotransformation. Microbial tryptophanase and decarboxylase enzymes convert dietary Trp into indole, tryptamine and other indole metabolites (indole-3 acetaldehyde, indole-3 acetic acid, indole-3 propionic acid, etc.). Mouse models of colitis reveal changes in serum levels of tryptophan [104]. An analysis of biopsies from UC and CD patients revealed a reduction in tissue kynurenine and increased levels of its metabolite 3-hydroxyanthranillic acid [105].

The aryl hydrocarbon receptor (AHR) is the main sensor for tryptophan metabolites and dietary ligands and is highly expressed in intestinal epithelial cells [106,107]. AHR regulates the expression of Reg3γ and S100A9 in the intestine [108]. AHR knockout mice also show reduced levels of tight junction proteins and enhanced gut permeability [10]. Epithelial AHR signalling also regulates the Wnt-β-catenin pathway to restrict excessive proliferation in the intestinal stem cells. The loss of AHR reduces the ability of the epithelium to repair and differentiate in response to tissue damage and contributes to an increase of inflammation-induced tumorigenesis [109]. GPR35 is well-expressed in the gastrointestinal tract and is predicted to be a receptor for Trp metabolites kynurenic acid and 2-oleoyllysophosphatidic acid. Single nucleotide polymorphisms (SNPs) in GPR35 are associated with higher risk for IBD and loss of GPR35 aggravates disease in a DSS-induced colitis model [94].

Indole-3 propionic acid (IPA) engages AHR to increase IL-10R1 expression on cultured epithelial cells as well as human organoid models. IPA treatment promotes barrier integrity and reduces the severity of DSS-induced colitis [110]. A seminal study in germ-free mice, monocolonized with either wild type *Clostridium sporogenes* or an IPA-synthesis mutant strain, led to an increased intestinal permeability, inflammation and immune response upon colonization with the mutant strain [38]. IPA can also be sensed by the xenobiotic sensor, pregnane X receptor (PXR). IPA supplementation partially reduced barrier permeability in germ free mice via PXR. While IPA alone is a weak agonist for human PXR, its binding it much stronger in the presence of indole [111].

*Lactobacillus* spp. can convert Trp to metabolites such as indole-3-aldehyde (IDA) which activate AHR signalling. IDA induces IL-22 secretion via AHR, which in turn promotes epithelial repair [112]. Furthermore, it has been shown that indole derivatives activate IL-10 production via AHR in aging mice to promote intestinal turnover, increase goblet-cell differentiation and strengthen the mucus barrier [113]. Scott et al. reported that the advantages of tryptophan-rich diets, as observed in a DSS-induced colitis model was due to the accumulation of metabolites such as indole-3 ethanol, indole-3 pyruvate and indole-3 aldehyde. These metabolites activate AHR, prevent the DSS-induced disassembly of adherens junction complexes to promote barrier integrity [114].

### 4.3. Bile Acid Metabolites

Primary bile acids (cholic acid and chenodeoxycholic acid) are synthesized in the liver, conjugated to taurine or glycine and released into the GI tract. Gut microbiota deconjugate these primary bile acids to liberate taurine or glycine. Deconjugated bile acids are further chemically modified by a variety of chemical processes including oxidation, dihydroxylation, esterification, etc., to produce secondary bile acids [115]. The enzymatic capacity for secondary bile acid formation is distributed across various bacterial species. For example, *B. fragilis* and *B. vulgatus* perform deconjugation, and dihydroxylation is carried out by *Clostridium* spp. and *Eubacterium* spp., while esterification is performed by *Bacteroides* spp., *Eubacterium* spp. and *Lactobacillus* spp. Around 95% of bile acids are reabsorbed in the terminal ileum by epithelial cells and are transported back to the liver [91].

Bile acids are detected by a variety of receptors, known collectively as bile acid-activated receptors (BAR), of which farnesoid X receptor (FXR) and G-protein bile acid receptor 1 (GPBAR1/TGR5/GP131) have beene well-studied. Lithocholic acid (LCA) is a ligand for the vitamin D receptor (VDR) and the pregnane X receptor detects both LCA and chenodeoxycholic acid (CDCA). Other receptors include sphingosine-1 phosphate receptor 2 (for LCA) and muscarinic receptor M3 (for LCA, deoxycholic acid). Many of these receptors are expressed in the liver, while intestinal epithelial cells are reported to express FXR, GPBAR1, PXR and VDR [115].

Expression of FXR is downregulated in IBD patients and SNPs in this gene are biomarkers for the severity of Crohn’s disease [116]. In mouse models, the deletion of FXR results in an increase in bile acid reabsorption by intestinal epithelial cells. Increased FXR deletion leads to increased barrier permeability and increases the severity of disease in colitis models [117]. Additionally, GPBAR-1 deletion in mice results in altered intestinal morphology, change in the architecture of tight junctions and increased intestinal permeability due to higher zonulin levels. GPBAR knockout mice are more susceptible to severe colitis upon exposure to DSS [118]. GPBAR also regulates the downstream effects of bile acids such as GI motility and regulates intestinal transit time [119]. The Vitamin D receptor is involved in IEC homeostasis. VDR suppresses IEC apoptosis, promotes the maintenance of the barrier and is protective against colitis [120]. VDR overexpression also upregulates tight junction proteins such as ZO-1, occludin, claudin-1 and claudin-15 and suppresses necroptosis in epithelial cells [121].

Both primary and secondary bile acids are regulators of barrier permeability. CDCA decreases occludin levels and increases permeability in human colon cell lines. However, the CDCA derivative, lithocholic acid, disrupts its effect. A similar antagonistic relationship was reported between ursodeoxycholic acid (UDCA) and deoxycholic acid (DCA) in the context of high-fat diet-induced gut permeability [10]. UDCA and LCA both prevent an increase in barrier permeability and inflammation in a DSS-induced colitis model. This effect is partly mediated by their inhibition of epithelial apoptosis [122]. UDCA and LCA also inhibit the epithelial inflammatory response and protect against DSS-induced colonic inflammation [123]. Additionally, UDCA promotes enterocyte migration via epidermal growth factor receptor- and cyclooxygenase-2-dependent mechanisms during injury and protects the barrier integrity [124].

### 4.4. Vitamins

Vitamins are essential micronutrients that cannot be synthesized by the body. Diet and de novo synthesis by commensal microbes are the main sources of vitamins. Vitamin (vit) synthesis pathways might be present within a single species or distributed across different species of bacteria. Thus, vitamin synthesis is a function of the microbial community as a whole [125].

Free thiamine (vit B1) and thiamine pyrophosphate (TPP) are both synthesized by bacteria. Bacterial TPP is absorbed by epithelial cells and used as a cofactor for ATP synthesis [126]. Bacteria such as *B. fragilis*, *Lactobacillus* spp., *Bifidobacterium* spp. and *Fusobacterium varium* possess the complete vit B1 biosynthetic machinery in the gut, while others such as *Faecalibacterium* spp. depend on other bacteria for the supply of this essential micronutrient [126]. Vitamin B2 (riboflavin) is essential in the TCA cycle and β-oxidation. Riboflavin is thought to promote immune cell differentiation and reactive oxygen species production. In a mouse model of chemically induced colitis, co-administration of riboflavin-producing bacterial species resulted in reduced tissue damage, microbial translocation and inflammatory response compared to riboflavin non-producers [127].

**Table 2:** Microbial metabolites and their effect on the intestinal epithelial barrier.

Metabolite	Microbial Source in the Gut	Mammalian Receptors	Effect on the Intestinal Epithelial Barrier
Short chain Fatty acids Butyrate, Acetate, Propionate, Lactate, Succinate, Valerate, etc.	Butyrate: *Clostridium* clusters I, III, IV, XI, XIVa, XV, and XVI [90] Acetate: *Bacteroides* spp. and *Prevotella* spp. Propionate: *Bacteroides* spp., *Veillonella* spp., *Dialister* spp. or *Ruminococcus* spp. [91]	Butyrate: GPR41, GPR109A, GPR65 (predicted) Acetate: GPR43 Propionate: GPR 41, GPR43 Lactate: GPR81 Succinate: GPR91 [92]	- Increase histone acetylation in IECs to modulate global gene expression [91] - Butyrate stimulates TGF-β secretion by IECs [92] - Butyrate inhibits proliferation of crypt stem cells via the transcription factor Foxo3 and promotes differentiation [91] - Butyrate utilization creates physiologic hypoxia and increases tight junction proteins such as Occludin and ZO-1 via hypoxia inducible factor (HIF). [92,96] - SCFAs promote antimicrobial production [93] - Lactate activates Wnt/β-catenin signalling in Paneth cells and Stromal cells to induce epithelial regeneration [94]
Bile acids Cholic acid, Lithocholic acid (LCA), Deoxycholic acid (DCA), Ursodeoxycholic acid (UDCA) etc.	*Bacteroides* spp., *Eubacterium* spp., *Lactobacillus* spp. *and Clostridium* spp. [115]	Farnesoid X receptor (FXR), GPBAR-1/TGR5, Pregnane X receptor (PXR), Vitamin D receptor (VDR), [115]	- FXR KO mice have higher intestinal permeability, high bacterial translocation and heightened bile acid reabsorption [117] - UDCA and LCA both inhibit epithelial apoptosis to limit DSS-induced barrier damage and inflammation [123] - DCA and LCA treatment in Caco-2 cells, reduce IL-1β induced IL-8 production [65] - UDCA promotes enterocyte migration [124]
Tryptophan metabolites Kynurenic acid, hydroxytryptamine, Indole derivatives	*Lactobacillus* spp., *Clostridium* spp. and *Bacteroides* spp.	GPR35 (predicted), Aryl hydrocarbon receptor (AHR), Pregnane X receptor (PXR) [106,111]	- Indole derivatives promote expression of anti-microbials [108] - Indoles regulate epithelial repair and differentiation [109,112] - Indoles promote IL-10 signalling to increase goblet cell differentiation and strengthen mucus barrier [113] - Indoles increase IL-10R1 expression on epithelial cells and reduces severity of DSS colitis [110] - Indoles prevent disassembly of adherens junction complexes during DSS colitis to maintain barrier integrity [114]

Niacin or nicotinic acid (vit B3) is synthesized from tryptophan by commensals. It is the ligand of GPR109a and can suppress inflammation and colitis via GPR109a signalling [128]. Niacin levels are lower in faecal samples of IBD patients [62,64,73]. In vitro, niacin was found to be anti-inflammatory and to reduce LPS-induced inflammatory response in Caco-2 cells [129]. In vivo, niacin upregulated prostaglandin D2 production, reduced epithelial cell death and improved epithelial healing to protect against DSS-induced colitis [130]. Pantothenic acid (vit B5) is a precursor to coenzyme A and plays an important role in the TCA cycle and β-oxidation. Bacteria such as *B. fragilis*, *P. copri* and *Ruminococcus* spp. possess the enzyme that is able to synthesize pantothenic acid from 2-dihydropantoate and β-alanine [126]. Vanin-1, an epithelial enzyme involved in the metabolism of pantothenic acid, is known to antagonize the peroxisome proliferator-activated receptor (PPAR) γ. The loss of vanin-1 resulted in reduced pro-inflammatory responses by IECs, which protected against chemically induced colitis in mice [131]. Pantothenic acid levels are also reduced in the faeces of IBD patients [62,64].

Biotin (vit B7) is a cofactor involved in amino acid and fatty acid metabolism. Additionally, biotin modifications on histone can regulate in cellular gene expression, including NF-κB [125]. Biotin is produced by commensal bacteria from malonyl CoA or pimelate and is absorbed by colonic IECs using the sodium-dependent multivitamin transporter (SMVT). *B. fragilis*, *P. copri*, Fusobacteria and Proteobacteria possess vit B7 synthetic machinery [126]. Some bacteria also co-operate in vit B7 synthesis. [132]. Biotin supplementation ameliorates DSS-induced colitis in mice by reducing NF-κB activation, pro-inflammatory cytokine production and intestinal permeability [133]. SMVT, which also transports pantothenate, is downregulated in biopsies of UC patients [133]. Mice harboring an intestine-specific knockout of SMVT display decreased biotin levels, abnormalities in the small intestine and develop spontaneous caecal inflammation [134].

Folate (vit B9) and tetrahydrofolate (THF) are essential in DNA and amino acid synthesis. *Lactobacillus* spp., some *Bifidobacteria* spp., *B. fragilis* and others synthesize THF, which is absorbed in the colon via the proton-coupled folate transporter [126]. Cobalamin (vit B12) is important for methionine synthesis. In the small intestine, dietary cobalamin is absorbed via the intrinsic factor, however the mechanism of absorption of bacterial cobalamin in the colon is currently unclear [135]. Neither folate deficiency nor cobalamin deficiency were seen to affect disease outcome in murine colitis models [136,137].

Vitamin K is generally known for its role in coagulation. While plants contain a form of vit K called phylloquinone, the form of vit K synthesized by gut microbes is called menoquinone [138]. IBD patients are known to be deficient in vit K, which is correlated to the loss of bacterial diversity in the gut [139]. However, the role for bacterially synthesized vitamin K in IBD remains unclear. Vitamin A occurs in the diet in the form of β-carotenes and retinyl esters and regulates antimicrobial production, cytokine secretion and IEC lineage determination in the gut [140]. Recently, the ability of gut microbes to synthesize vitamin A derivative, retinoic acid, has been reported [141]. The way in which this gut derived retinoic acid affects the pathogenesis of IBD still needs to be investigated.

### 4.5. Other Metabolites

#### 4.5.1. Medium and Long Chain Fatty Acids

Medium chain fatty acids are detected by GPR40 and GPR84, while long chain fatty acids are detected by GPR40 and GPR120. Long chain fatty acids such as linoleic acid improve the barrier function via GPR40 and the extracellular signal-regulated kinase signalling pathway [94]. MCFAs and LCFAs both have pro-inflammatory effects on epithelial and immune cells [142]. This results in the downregulation of tight junction protein levels and impairment of the intestinal barrier. Additionally, high-fat diets result in increased bile acid secretion, which in turn negatively regulates the barrier integrity [143].

#### 4.5.2. Sulfur-Containing Metabolites

Sulfur-containing metabolites are crucial for health. Methionine (Met), carbon disulfide, dimethyl trisulfide, dimethyl disulfide and taurine are the common sulfur-containing metabolites in healthy human faeces [144]. Taurine and hydrogen sulfide are both reported to be higher in IBD patients [145]. Taurine activates inflammasome complexes to increase IL-18 expression through intestinal epithelial cells [146]. Taurine has been shown to have antioxidant effects and to protect the gut barrier from oxidative stress-induced injury by upregulating the expression of tight junction proteins claudin-1, ZO-1 and occludin [147]. Hydrogen sulfide also presents antioxidant, anti-inflammatory and anti-apoptotic activity in the intestine. It was found that H_2_S is able to protect Caco-2 cells from cytokine-induced inflammation and barrier disruption by blocking NF-κB activation. Moreover, H_2_S is also known to stabilize hypoxia-inducible factor- 1α resulting in protection during experimental colitis [148].

#### 4.5.3. Polyamines

Polyamines, such as putrescine, spermidine and spermine are produced by *Bacteroides* spp., *Fusobacterium* spp. and *E. coli* in the gut via arginine metabolism. Polyamines are important for epithelial proliferation and repair. Polyamines promote barrier integrity by increasing the transcription of E-cadherin [91]. Spermine inhibits IL-18 production which is involved in epithelial repair and the barrier function [146]. In a mouse model of high-fat diet, spermidine supplementation improved barrier permeability, increased the number of mucus-producing goblet cells and reduced intestinal inflammation [149,150]. In cell line models, spermidine increased the expression of tight junction and autophagy-related markers while decreasing apoptosis-related markers [150]. Additionally, monocolonization experiments in germ free mice with wild-type or polyamine-synthesis deficient *E. coli* strains revealed that commensal-derived polyamines are taken up by colonocytes. Commensal polyamines promoted epithelial renewal and ameliorated disease severity in a DSS-induced colitis model [151].

#### 4.5.4. Polyphenol Metabolites

Polyphenols, such as tannins and flavones, in the diet are metabolized by gut bacteria into phenolic derivatives that influence intestinal function. For example, urolithins are produced by dietary ellagic acid. Urolithin A treatment upregulates tight junction proteins in an AHR-dependent pathway to improve gut barrier integrity and reduces the severity of DSS-induced colitis [10]. Lignan derivatives, namely equol and enterolactone, also protect against barrier dysfunction induced by inflammatory cytokines such as TNFα and IL-6 [10]. The barrier-protective function of polyphenol might be mediated via the inhibition of NF-κB signalling, or the upregulation of tight junction proteins including ZO-1 and occludin [152].

## 5. Future Perspectives

Metabolomics investigations have provided crucial information about the changing metabolic landscape in the gut of IBD patients. The sheer chemical complexity of this metabolic landscape presents a challenge in the field. To identify the scope of this complexity, it is necessary to use a combination of metabolomics pipelines combined with techniques that provide high resolution and sensitivity [37,45]. A key challenge is presented by the dark matter in metabolomics, whereby less than 2% of the features in spectra are annotated [153]. The use of standard libraries, while resource-intensive, can help to overcome this hurdle. Additionally, studies have also used co-variate analysis and a “guilty-by-association” approach to gain insight into the functional role of unidentified metabolites [63].

Studies in pre-clinical models, and clinical investigations have established microbial metabolites as key regulators of the intestinal barrier. In contrast to genetic susceptibility, the dynamic nature of metabolites makes them vulnerable to manipulation via therapeutic strategies. The design of these strategies requires a comprehensive understanding of the dietary and microbial influence on metabolite production [73]. This has led to a shift towards multi-omic investigations in IBD patients. A combination of metabolomic and metagenomic data allows for an investigation of the physiological relationships between microbes and metabolites [63,64]. Using a dual omics approach, Santoru et al. uncovered that the bacterial genera *Faecalibacterium* and *Oscillospira* (both downregulated in IBD) negatively correlate with polyamine levels and positively correlate with 5β-coprostanol levels in the faecal samples of IBD patients. Decreased *Faecalibacterium* was also associated with lower level of vitamins in CD and UC patients. The *Flavobacterium* genus was found to negatively correlate with TMAO levels but positively correlated with phosphatidylcholines, 2-hydroxy-3methyvaleric acid, citric acid and methylamine [62]. Interestingly, this study did not observe the expected reduction in Firmicutes in the gut microbiota of IBD patients, though an increase in Proteobacteria was observed. Franzosa et al. analyzed metabolite–microbe interactions and observed a bias towards concordant (same direction) associations in their study, with discordant associations making up only 2% of all significant associations. They observed a positive correlation between lactic acid and *Pediococcus acidilactici*. LCFAs such as docosapentaenoic acid (DPA) and eicosatrienoic acid (ETA) (both elevated in CD and UC patients) had a negative correlation with control-associated species such as *Eubacterium ventriosum* and a positive association with IBD-associated species such as *R. gnavus* [63].

Systems biology approaches have been particularly useful in combining microbial genetic potential with dietary inputs to predict the metabolic outputs in a system. A recent study by Heinken et al. used existing metabolomic and metagenomic data to develop a pipeline to predict the metabolic profile of complex microbial communities in IBD patients. Their model predicted that dysbiotic IBD microbiota had an increased potential for amino acid synthesis and the secretion of metabolites such as lactate, putrescine and hydrogen sulfide. Alongside this, these communities had a reduced potential for the secretion of branched chain fatty acids and vitamin B3 [154]. Such predictive models provide a valuable tool for uncovering previously unknown or unappreciated relationships between gut microbes and metabolites in IBD. Additionally, artificial intelligence (AI)-driven approaches have been proved to be useful in unravelling host-signalling pathways that regulate barrier function. Sahoo et al. recently implemented an AI approach to identify potential barrier protective nodes that could be targeted for therapeutic benefit. They further used in vitro and in vivo models to provide proof of concept of therapeutic success [155].

The translational potential of metabolite-based approaches is hampered by a lack of understanding of the physiological role of metabolites. High-throughput screens have provided an effective strategy for uncovering hitherto unknown biological interactions and functional effects. In vitro screens have been used to identify physiological receptors for microbial metabolites. Availability of in vitro GPCR libraries has allowed screening of human gut microbes as well as their metabolites to identify novel interactions and potential physiological effects [156,157]. Recently, Grosheva et al. screened libraries of known microbial metabolites, secreted proteins, and drugs against a high throughput in vitro system to identify stabilizers and disruptors of the epithelial barrier. They identified that acetyl-proline, spermine and putrescine have a disruptive effect on the barrier while taurine, tryptamine and L-homo-serine stabilize barrier integrity. These in vitro observations were further validated in vivo using a mouse model of DSS-induced colitis. In vivo, putrescine disrupted the gut barrier, but co-administration of taurine could ameliorate these effects [158]. These results illustrate how minor changes in the balance of metabolites in the gut could dynamically modulate barrier functions and hence disease susceptibility.

Probiotics and prebiotics have been employed for the treatment of IBD with mixed success [159,160,161]. Probiotics (single or mixture) as well as synbiotics (probiotics + prebiotics) have shown promise in animal models of IBD [162]. Predominant strains such as *Lactobacillus* GG (LGG), *L. johnsonii*, *Saccharomyces boulardii*, *E. coli* strain Nissle 1917 and *B. longum* have also been evaluated for their therapeutic potential in human trials. Some randomized control trials (RCTs) with probiotics and synbiotics in CD patients have shown sporadic success, however, larger trials have failed to reveal benefits of probiotics in ameliorating disease severity or promoting remission [163]. Probiotics and synbiotics have produced greater success in the treatment of UC. Multiple RCTs, using the probiotic VSL#3 (mixture of 8 different *Bifidobacterium*, *Lactobacillus* and *Streptococcus* spp.), showed that VSL#3 along with low-dose standard therapy achieved similar rates of remission as medium-dose therapy alone [164,165]. RCTs comparing *E. coli* strain Nissle 1917 and LGG to standard therapy, respectively, did not find any statistically significant difference between them [166,167]. This suggests that probiotics might be as effective as standard therapy in maintaining remission in UC patients. However, larger and better-designed RCTs are required before definitive conclusions can be drawn about the benefits of prebiotics and probiotics in IBD [168]. The integration of a metabolomics analysis into these RCTs can help uncover prognostic markers of therapeutic success and help inform a better design of live-biotherapeutic-based treatment regimens. Another challenge presented for the efficacy of live biotherapeutics-based therapies is an incomplete understanding of the dynamic relationships between microbes, diet, and inflammation. Metabolomic approaches can provide insight into microbial functions that need to be corrected or reintroduced into the dysbiotic gut of IBD patients. A personalized design of prebiotic and probiotic regimens is required to ensure that the biotherapeutic agent can survive, stably colonize, and produce metabolites of interest in the inflamed gut [169].

In addition to metabolites, microbes produce secreted and surface-factors that exhibit an immunomodulatory function, collectively called postbiotics. Postbiotics are defined as a preparation of inanimate microorganisms and/or their components that confers a health benefit on the host [170]. It includes any soluble factor that is produced by the metabolic activity of a probiotic organism, including microbial polysaccharides, sphingolipids, proteins or phages that could have immunomodulatory effects [91]. A detailed overview of postbiotics and their beneficial effects has been provided in other valuable reviews [91,171,172]. The contribution of postbiotics to the beneficial effects of fermented foods or commonly used probiotic organisms, especially in the context of barrier function, is still not fully appreciated [173,174]. Postbiotics have a better safety profile compared to live probiotic organisms and are more amenable from a scale-up and reproducibility perspective [172]. They have the potential to interrupt the vicious cycle between gut inflammation and dysbiosis and promote the reestablishment of the indigenous microbiota. Clinical trials are required to reveal the therapeutic potential of postbiotics for IBD.

## 6. Conclusions

**Figure 1:**
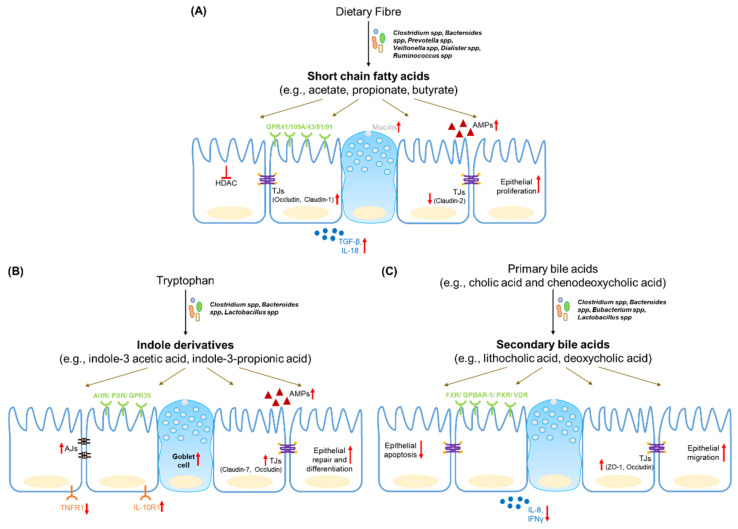
Schematic summary of the regulation of the intestinal epithelium by the microbial metabolites, short chain fatty acids (**A**), tryptophan metabolites (**B**) and bile acids (**C**). Dietary or host compounds are metabolized by commensal bacterial species to produce cognate metabolites. Microbial metabolites are absorbed and/or detected by receptors on intestinal epithelial cells (marked in green) to mediate downstream effects. Red arrows highlight regulation of major barrier determinants such as tight junctions (TJs), adherens junctions (AJs), cytokine signalling, epithelial proliferation/differentiation, histone deacetylase (HDAC), anti-microbial peptides (AMPs) and mucins.

To summarize, microbial metabolites form a crucial link in host–microbe communication and are key regulators of barrier function and intestinal homeostasis (Figure 1). Metabolomics revealed the dynamic changes in the gut metabolite profiles of IBD patients. These changes in metabolites are of diagnostic and prognostic value, and have the potential to improve the clinical care provided to IBD patients. Multi-omic approaches have provided valuable information with regard to the relationships between microbes and gut metabolites. Centering metabolites as functional mediators in a diet-microbe-host dialogue can allow for an improved stratification of IBD patients, and can help to inform the rational design of personalized nutritional and biotherapeutic approaches for IBD treatment [175].

## Data Availability

Not applicable.
